# Glaucoma incidence risk in a cohort of Mayak PA workers occupationally exposed to ionizing radiation

**DOI:** 10.1038/s41598-019-48915-6

**Published:** 2019-08-28

**Authors:** Evgeny V. Bragin, Tamara V. Azizova, Maria V. Bannikova, Evgeniya S. Grigoryeva, Nobuyuki Hamada

**Affiliations:** 1 Southern Urals Biophysics Institute (SUBI), Ozyorskoe Shosse 19, Ozyorsk, Chelyabinsk Region 456780 Russia; 2 Radiation Safety Research Center, Nuclear Technology Research Laboratory, Central Research Institute of Electric Power Industry (CRIEPI), 2-11-1 Iwado-kita, Komae, Tokyo 201-8511 Japan

**Keywords:** Epidemiology, Glaucoma

## Abstract

Glaucoma is a major cause of visual impairment, and secondary glaucoma manifested as neovascular glaucoma has long been known to occur following high-dose fractionated radiation therapy. In contrast, little is known as to whether ionizing radiation exposure causes primary glaucoma, except that a single study in Japanese atomic bomb survivors has reported a significantly increase risk. Therefore, the effect of lower dose and lower dose rate remains unclear. Here we report that in Russian Mayak Production Association workers occupationally exposed to chronic radiation for prolonged periods, incidence of total primary glaucoma and primary open-angle glaucoma is significantly associated with various non-radiation factors (sex, attained age, and cataract diagnosed prior to glaucoma), but neither with cumulative dose from external γ-rays nor with cumulative neutron dose nor with the unweighted sum of cumulative γ and neutron doses. The present results suggest for the first time that chronic radiation exposure does not cause primary glaucoma, although the analyses need to be made in other cohorts exposed at various dose and dose rate.

## Introduction

Glaucoma is a significant medical and social issue in many countries including Russia and Japan. Glaucoma reduces the quality of life of a patient, and represent one of the major causes of blindness worldwide^1^. Glaucoma covers a large group of ocular diseases sharing similar clinical, pathogenic and pathomorphological features. When the eyes suffer from glaucoma, the intraocular pressure (IOP) permanently or occasionally exceeds a level of tolerance, distinctive optic disk (OD) changes and ganglion cell degeneration (glaucomatous optic neuropathy) develop, leading to associated ocular dysfunction. Glaucoma may develop at different ages starting from birth, but its incidence increases with age^1,2^. Glaucoma is classified into specific types: congenital, primary glaucoma, and secondary glaucoma. Congenital glaucoma is related to fetus disorders and birth injuries. Primary glaucoma has a multifactorial nature and is associated with age-related (involutional) changes in the eyes. Primary glaucoma is classified into primary open-angle glaucoma (POAG) and primary angle-closure glaucoma (PACG), the former being further classified into normal-tension glaucoma (NTG) and high-tension glaucoma (HTG). Secondary glaucoma (e.g., neovascular glaucoma) is caused by another ocular or somatic pathology. Among risk factors for glaucoma are elderly age, heredity (excess disease incidence associated with familial group), vascular factors (increased or decreased blood pressure, atherosclerosis), and endocrine disorders (diabetes mellitus, abnormal glucocorticoid metabolism)^3,4^.

Local ocular or head and neck therapeutic irradiation at high doses (>about 40–50 Gy) is known to increase risk of secondary glaucoma (mostly, neovascular)^5–11^. Studies of atomic bomb survivors acutely exposed to radiation at high dose rates demonstrated increased risk only for primary open angular normal tension glaucoma only^12,13^. A recently published study of US radiologic technologists (USRT) showed no significant association of glaucoma with occupational radiation at low doses^14^. Therefore, a question of whether ionizing radiation induces glaucoma development remains open and other radiation exposed cohort studies are needed to replicate the observed results. This study aims to estimate primary glaucoma incidence risk in Mayak PA workers occupationally exposed to chronic radiation at low dose rates for prolonged periods.

## Methods

### Study population and follow-up

The Mayak Production Association (PA) is the first large-scale Russian nuclear enterprise. It is located in the Southern Urals close to the city of Ozyorsk and started its operation in 1948.

The study cohort included all workers of the enterprise first employed at one of the main facilities (reactors, radiochemical and plutonium production plants) in 1948–1982 (the mean calendar year of employment was 1959.4 ± 9.8 years), who were followed up until the end of 2008: there were a total of 22,377 individuals (of whom 25.4% were females). As for start of employment, the mean and standard deviations of age was 24.9 ± 7.5 years (min–max: 17–65 years). Duration of employment at the Mayak PA was 17.9 ± 14.1 years and ranged from 1 month up to 60 years. Only 4.70% of workers were employed at the Mayak PA less than 1 year.

The cohort follow-up started from the date of first employment at one of the main facilities and ended at the earliest of the following dates: disease diagnosis, date of death, 31 December 2008 for alive workers still residing in Ozyorsk (‘residents’), date of ‘the last medical information’ for workers-residents with an unknown vital status and for those who had left Ozyorsk (‘migrants’). The main characteristics of the study cohort are presented in Tables 1 and S1. By the end of the follow-up period, a vital status was available for 95.0% of the study cohort members with 53.5% deceased and 46.5% alive.

**Table 1:** Study cohort characteristics.

Characteristics	Males	Females	Both
Number of workers included in the cohort	16,688	5,689	22,377
Total primary glaucoma cases	315	161	476
POAG cases	307	154	461
PYR (Total primary glaucoma incidence)	383,262	160,974	544,236
PYR (POAG incidence)	383,188	160,874	544,062
Mean age at diagnosis of total primary glaucoma (SD), years	64.6 (8.5)	69.0 (8.8)	66.1 (8.8)
Mean age at diagnosis of POAG (SD), years	64.6 (8.4)	69.4 (8.3)	66.2 (8.7)
Migrated from Ozyorsk as of 31 December 2005	7,198 (41.1%)	2,014 (35.4%)	9,212 (41.1%)
Vital status known as of 31 December 2008, of them:	15,870 (95.1%)	5,436 (95.6%)	21,267 (95.0%)
Died	8,954 (56.4%)	2,417 (44.5%)	11,371 (53.5%)
Alive	6,916 (43.6%)	3,019 (55.5%)	9,896 (46.5%)
Incidence data available	16,233 (97.3%)	5,530 (97.2%)	21,763 (97.3%)
Smoking status available, of them:	15,636 (93.7%)	5,270 (92.6%)	20,906 (93.4%)
Known data on qualitative parameters of smoking	11,064 (70.8%)	5,172 (98.1%)	16,236 (77.7%)
Data on alcohol consumption available	14,857 (89.0%)	5,173 (90.9%)	20,030 (89.5%)
Data on hypertension available	15,110 (90.5%)	4,984 (87.6%)	20,094 (89.8%)
Data on body mass index	13,732 (82.3%)	4,660 (81.9%)	18,392 (82.2%)
Mean age at first employment (SD), years	24.1 (7.1)	27.3 (8.0)	24.9 (7.5)
Mean calendar year at first employment (SD), calendar years	1959.8 (9.8)	1958.0 (10.4)	1959.4 (9.8)
Mean duration of employment at the Mayak PA (SD), years	18.3 (14.8)	17.4 (12.8)	17.9 (14.1)
Mean age at death for workers known to have died (SD), years	60.2 (13.6)	68.5 (12.4)	62.00 (13.8)
Mean age of workers known to be alive (SD), years	66.5 (10.1)	74.8 (9.3)	68.8 (11.5)
Mean age as of migration date (SD), years	31.2 (10.2)	34.2 (11.9)	31.6 (10.3)
Mean cumulative brain absorbed dose from external γ-rays (SD), Gy^a^	0.46 (0.67)	0.36 (0.56)	0.44 (0.65)
Workers without neutron exposure	13,213 (79.2%)	5,081 (89.3%)	18,294 (81.8%)
Workers with neutron exposure	3,475 (20.8%)	608 (10.7%)	4,083 (18.2%)
Mean cumulative brain absorbed dose from neutrons (SD), Gy^b^	0.0016 (0.0043)	0.0016 (0.0050)	0.0016 (0.0044)
The unweighted sum of mean cumulative brain absorbed γ + neutrons dose (SD), Gy^a^	0.46 (0.67)	0.37 (0.56)	0.44 (0.65)

*Notes:* POAG, primary open-angle glaucoma. PYR, person years at risk. SD, standard deviation. PA, production association.

^a^For all workers.

^b^For workers with neutron exposure.

All glaucoma cases were retrospectively coded according to the International Classification of Diseases, ninth revision (ICD-9)^15^. All (total 634) cases of glaucoma (ICD-9: 365.0–365.9) diagnosed in members of the study cohort were identified using the medical and dosimetry database ‘Clinic’^16^ regardless of its type. Glaucoma was diagnosed by a qualified ophthalmologist based on generally accepted criteria (complaints, measurements of IOP, eye fundus examination, visual field tests, and gonioscopy). Of 634 cases, 476 cases (75.1%) were primary glaucoma, comprising 461 cases (96.8%) of POAG and 15 cases (3.2%) of PACG. The study outcomes included these 476 cases of primary glaucoma, because secondary glaucoma (158 cases) occurs as complications accompanying an ocular or somatic pathology.

### Dosimetry

For analyses, absorbed doses from external γ-rays and neutrons were used based on the Mayak Worker Dosimetry System 2008 (MWDS–2008)^17^. MWDS–2008 provides absorbed doses in 18 organs and tissues (lungs, red bone marrow, breast, bladder, kidney, skin, stomach, gonads (ovaries/testicles), thyroid, uterus, esophagus, bone surface, skeleton, liver, small intestine, large intestine, spleen), but eye dose is not available. This study therefore used individual brain absorbed doses from external γ-rays and neutrons^18^. It should be noted that there is no considerable difference among types of organ absorbed doses from external radiation. Mean cumulative brain absorbed dose from external γ-rays was 0.46 ± 0.67 Gy in males and 0.36 ± 0.56 Gy in females. Only 4083 Mayak PA workers (18.2%) were exposed to neutrons. For these workers, the mean cumulative brain absorbed external radiation dose from neutrons was 0.0016 ± 0.0043 Gy in males and 0.0016 ± 0.0050 Gy in females. For those workers who had not been exposed to neutrons, doses from neutrons were not estimated and in the present study they were considered as ‘unmeasured 0.00’. The mean unweighted sum of cumulative γ and neutron brain absorbed doses was 0.46 ± 0.67 Gy in males and 0.37 ± 0.56 Gy in females. Distribution of the study cohort workers by radiation dose is presented in Table S1.

### Statistical analysis

To perform analyses of the data on the study cohort, the statistical techniques and methods were consistent with previous studies^19–21^. The dataset for analyses was restricted to a period of residence in Ozyorsk, because information on diseases, the results of annual eye examinations and non-radiation factors was unavailable for migrants after they had left the city. This study excluded 43 workers with acute radiation sickness due to acute single high dose-rate γ + neutron exposure, and also excluded 614 workers with missing medical information due to lost medical charts and 77 workers with glaucoma diagnosed prior to employment at the Mayak PA.

As in the previous studies^19–21^, the analysis provided estimates of relative risk (RR) for categories designated for one or more variables while adjusted for other variables. RR was computed based on maximum likelihood using the AMFIT module of the EPICURE software^22^. In order to test a statistical significance, 95% confidence intervals (CIs) for RRs, and *p* values were computed using likelihood-based techniques integrated in the AMFIT module.

In consistence with the previous studies^19–21^, categorical analyses and dose-response analyses were run using Poisson regression in the AMFIT module of EPICURE software^19^. Excess relative risk per unit dose (ERR/Gy) was modeled by a linear trend with dose from external γ-rays, neutron dose and unweighted γ + neutron dose lagged for 5 years including adjustment (via stratification) for non-radiation factors (sex, attained age (<20, 20–25, …, 80–85, ≥85 years), birth cohort (<1910, 1910–1919, 1920–1929, 1930–1939, 1940–1949, ≥1950), hypertension (hypertension-free, hypertension, unknown), body mass index (BMI) (below normal, normal, above normal, obese, unknown), diabetes mellitus (DM) (free from DM, diagnosed with DM) and neutron dose for γ-ray exposure association analysis and vice versa.

Analysis of glaucoma incidence associated with dose from external γ radiation adjusted for neutron dose was set as the default analysis. It considered the whole cohort. The neutron dose adjustment was included using stratification, and the neutron dose was treated as a categorical variable. In consistence with the previous studies^19–21^, workers who were assumed not to have been exposed to neutrons were not excluded from the analyses; they were included in the ‘unmeasured 0.00’ category. Analyses of the linear trend with neutron dose and unweighted γ + neutron dose were conducted as sensitivity analyses. The analysis of the linear trend with neutron dose was restricted to workers with measured (or known, or estimated) doses from neutrons (4083 workers). The analysis of the linear trend with unweighted γ + neutron dose considered the whole cohort. To estimate the unweighted γ + neutron dose, the unmeasured neutron dose was regarded as equal to 0.00.

In addition, sensitivity analyses were performed to investigate effects of dose lagging (0, 10, 15, 20-year lag periods) and additional non-radiation factors, such as diagnosed cataract or cataract removal surgery prior to a report of glaucoma, alcohol consumption and smoking status, smoking index (<10, 10–20, >20 pack-years) rather than smoking status parameter on the observed risk estimates in relation to external radiation exposure.

Using log-linear modifications of excess relative risk per unit dose we tested whether such factors as sex, age at first employment and attained age of workers could modify the radiation risk of glaucoma incidence. All p-values were for two-sided test. Differences were judged to be significant at *p* < 0.05.

Since hypertension, BMI and DM are standard risk factor for glaucoma, all these factors were considered in the default analysis (with 5-year lag) via stratification. We excluded these variables as well as the variable of neutron dose, one by one thus investigating the effect of removing these variables from the model.

Definitions and categorizations of risk factors considered in this study are provided in details in Supplementary Section.

Information about whether a worker had a diagnosis of DM and cataract and whether he/she underwent cataract surgery was available for every worker of the study cohort. Cases with cataract and cataract removal surgery were included in the analyses if they were registered before glaucoma was diagnosed in a worker.

An additional analysis was performed to provide odds ratios of various risk factors for primary glaucoma and POAG. The analysis was based on a logit regression and is described in details in Supplementary Section.

### Ethical statement

The study was reviewed and approved by the SUBI Institutional Review Board who confirmed that no signed consents were needed from members of the study cohort, because the present record-based epidemiological study did not require any contact with cohort members.

## Results

By the end of the follow-up period, 476 cases of primary glaucoma were registered over 544,236 person-years of follow-up. Of these, RR of glaucoma incidence was calculated each for primary glaucoma and POAG, but not for PACG due to a small number of cases (15 cases).

Risk analysis started with assessment of its associations with known non-radiation factors promoting glaucoma (sex, attained age, smoking, alcohol consumption, cataract and cataract removal surgery, birth cohort, calendar period of diagnosis date, age at first employment, hypertension, BMI, and DM). Tables 2 and S2 summarize the results of the analysis of glaucoma incidence in relation to non-radiation factors.

**Table 2:** Risk analysis results for primary glaucoma and POAG incidence in relation to non-radiation factors.

Factors		Total primary glaucoma			POAG		
		RR (95% CI)	Number of cases	*p* value	RR (95% CI)	Number of cases	*p* value
***RR females vs. males***							
Males	1 (reference)	315	—	1 (reference)	307	—	
Females	0.68 (0.56, 0.82)	161	<0.001	0.66 (0.54, 0.81)	154	<0.001	
***RR for various age groups (compared to 65–70 years old group)***							
Males	<40	—	0	—	—	0	—
	40–45	0.02 (0.01, 0.04)	5	<0.001	0.01 (0.01, 0.03)	4	<0.001
	45–50	0.04 (0.02, 0.08)	12	<0.001	0.04 (0.02, 0.08)	12	<0.001
	50–55	0.10 (0.06, 0.15)	22	<0.001	0.10 (0.06, 0.16)	22	<0.001
	55–60	0.27 (0.18, 0.39)	46	<0.001	0.27 (0.18, 0.39)	45	<0.001
	60–65	0.55 (0.39, 0.76)	67	<0.001	0.55 (0.40, 0.77)	66	<0.001
	65–70	1 (reference)	81	—	1 (reference)	79	—
	70–75	1.46 (1.03, 2.06)	55	0.032	1.45 (1.01, 2.05)	53	0.040
	75–80	1.10 (0.63, 1.81)	18	>0.50	1.14 (0.65, 1.87)	18	>0.50
	80–85	1.76 (0.77, 3.49)	8	0.140	1.59 (0.66, 3.28)	7	0.252
	≥85	1.00 (0.06, 4.68)	1	>0.50	1.04 (0.06, 4.83)	1	>0.50
Females	<40	—	0	—	—	0	—
	40–45	0.05 (0.01, 0.15)	3	<0.001	0.02 (0.01, 0.08)	1	<0.001
	45–50	0.07 (0.02, 0.18)	4	<0.001	0.07 (0.02, 0.17)	4	<0.001
	50–55	0.11 (0.04, 0.25)	6	<0.001	0.07 (0.02, 0.18)	4	<0.001
	55–60	0.10 (0.03, 0.23)	5	<0.001	0.10 (0.03, 0.22)	5	<0.001
	60–65	0.66 (0.40, 1.09)	29	0.107	0.62 (0.37, 1.02)	28	0.062
	65–70	1 (reference)	34	—	1 (reference)	35	—
	70–75	1.58 (0.99, 2.53)	37	0.057	1.50 (0.93, 2.40)	36	0.093
	75–80	2.23 (1.35, 3.67)	31	0.001	1.96 (1.17, 3.25)	28	0.010
	80–85	2.20 (1.01, 4.38)	10	0.033	2.38 (1.13, 4.65)	11	0.015
	≥85	1.81 (0.29, 6.25)	2	0.428	1.85 (0.29, 6.42)	2	0.410
***RR for workers diagnosed with vs. without hypertension***							
Males	Unknown	0.78 (0.47, 1.21)	19	0.294	0.75 (0.45, 1.19)	18	0.250
	Hypertension-free	1 (reference)	247	—	1 (reference)	243	—
	Hypertension	0.99 (0.72, 1.34)	49	>0.50	0.95 (0.68, 1.29)	46	>0.50
Females	Unknown	0.77 (0.41, 1.31)	13	0.360	0.81 (0.43, 1.38)	13	0.464
	Hypertension-free	1 (reference)	129	—	1 (reference)	123	—
	Hypertension	1.00 (0.60, 1.58)	19	>0.50	0.99 (0.58, 1.59)	18	>0.50
***RR for workers with increased or decreased BMI in relation to workers with normal BMI***							
Males	Unknown	0.97 (0.67, 1.36)	36	>0.50	0.96 (0.66, 1.37)	35	>0.50
	BMI < 18.5 kg m^−2^	0.80 (0.20, 2.09)	3	>0.50	0.81 (0.20, 2.13)	3	>0.50
	BMI 18.5–24.9 kg m^−2^	1 (reference)	232	—	1 (reference)	227	—
	BMI 25–29.9 kg m^−2^	0.77 (0.54, 1.07)	39	0.135	0.75 (0.52, 1.04)	37	0.101
	BMI ≥ 30 kg m^−2^	1.09 (0.39, 2.36)	5	>0.50	1.11 (0.40, 2.42)	5	>0.50
Females	Unknown	0.83 (0.50, 1.32)	21	0.448	0.80 (0.48, 1.29)	20	0.383
	BMI < 18.5 kg m^−2^	—	0	—	—	0	—
	BMI 18.5–24.9 kg m^−2^	1 (reference)	83	—	1 (reference)	82	—
	BMI 25–29.9 kg m^−2^	1.08 (0.74, 1.55)	44	>0.50	1.00 (0.67, 1.44)	40	>0.50
	BMI ≥ 30 kg m^−2^	1.21 (0.64, 2.10)	13	>0.50	1.13 (0.59, 2.00)	12	>0.50
***RR for workers with vs. without DM***							
Males	Free from DM	1 (reference)	293	—	1 (reference)	285	—
	Diagnosed with DM	1.35 (0.85, 2.03)	22	0.182	1.39 (0.87, 2.10)	22	0.140
Females	Free from DM	1 (reference)	145	—	1 (reference)	139	—
	Diagnosed with DM	1.26 (0.72, 2.06)	16	0.380	1.21 (0.68, 2.00)	15	0.488
***RR for workers diagnosed with vs. without cataract***							
Males	Free of cataract	1 (reference)	89	—	1 (reference)	85	—
	Diagnosed with cataract	12.09 (9.08, 16.23)	226	<0.001	12.56 (9.40, 16.95)	222	<0.001
Females	Free of cataract	1 (reference)	38	—	1 (reference)	32	—
	Diagnosed with cataract	8.43 (5.54, 13.19)	123	<0.001	9.45 (6.09, 15.16)	122	<0.001

*Notes:* Bold font, *p < *0.05. BMI, body mass index. CI, confidence interval estimated using the profile likelihood. DM, diabetes mellitus. POAG, primary open-angle glaucoma. RR, relative risk. *p* values are given, assessed via the Wald statistics.

RR for incidence of both primary glaucoma and POAG was significantly lower in females than in males. The incidence risks of primary glaucoma and POAG increased significantly with attained age of workers of both sexes. Incidence of primary glaucoma and POAG was not significantly associated with a calendar period of first employment except for the significantly lower incidence among females hired in 1969–1972 (RR = 0.30 (95% CIs: 0.05, 0.97) and 0.31 (95% CIs: 0.05, 0.99), respectively), which could likely be explained by a small number of cases falling into this group (2 cases). Incidence of primary glaucoma and POAG was significantly associated with certain calendar periods for workers of both sexes. RR for incidence of primary glaucoma and POAG was insignificant in groups of workers by age of first employment at the Mayak PA except for decreased risk in males hired at age 25–30 years compared to those hired before age 20 years. None of smoking status, alcohol consumption status, smoking index and BMI affected incidence of primary glaucoma and POAG in both males and females. Incidence of primary glaucoma and POAG was notably higher in workers diagnosed with cataracts (preceding glaucoma) than those without cataracts: RR in males was 12.09 (95% CIs: 9.08, 16.23) and 12.56 (95% CIs: 9.40, 16.95), respectively; RR in females was 8.43 (95% CIs: 5.54, 13.19) and 9.45 (95% CIs: 6.09, 15.16), respectively. Increased albeit insignificant risk estimates were demonstrated for incidence of primary glaucoma and POAG among workers after cataract removal surgery as well as those with diagnosed DM.

**Figure 1:**
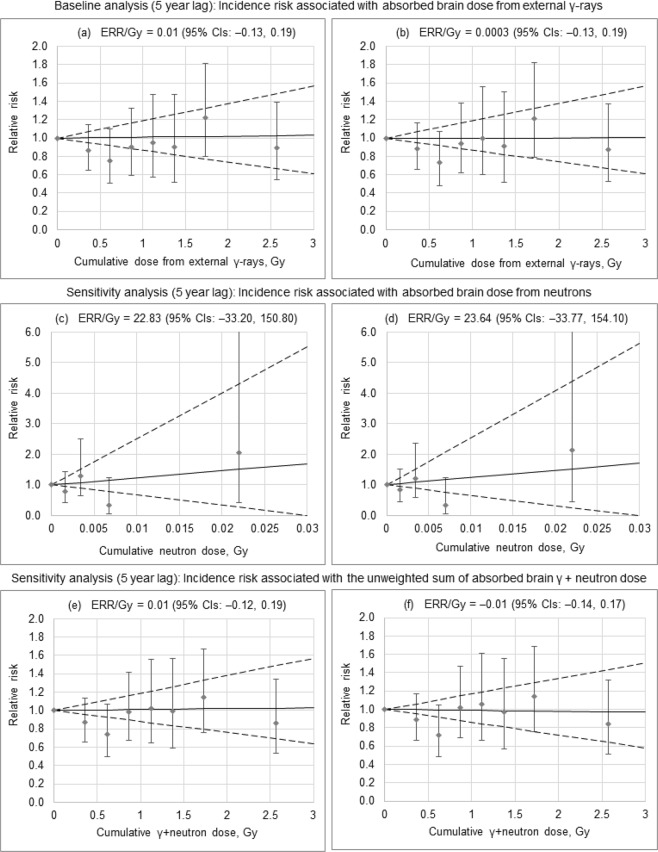
Primary glaucoma (**a**,**c**,**e**) and POAG (**b**,**d**,**f**) incidence by cumulative dose from external γ-rays (**a**,**b**), neutron dose (**c**,**d**) and γ + neutron dose (**e**,**f**).

Next, RRs were analyzed for incidence of primary glaucoma and POAG in relation to cumulative dose from external γ-rays, neutrons and γ + neutron exposure. RRs for incidence of primary glaucoma and POAG resulting from all these analyses were insignificant in all dose groups (Table 3). Using a linear model, the primary glaucoma incidence risk, in particular, risk of POAG, was analyzed in relation to external γ-dose (Table 4, Fig. 1). Incidence of primary glaucoma and POAG was not significantly associated with cumulative dose from external γ-rays, regardless of adjustment for neutron dose with ERR/Gy of 0.01 (95% CIs: −0.13, 0.19) and 0.0003 (95% CIs: −0.13, 0.19) when adjusted and 0.01 (95% CIs: −0.12, 0.20) and 0.01 (95% CIs: −0.13, 0.19) when unadjusted, respectively. Moreover, no significant associations were found with cumulative neutron dose and with cumulative γ + neutron dose for either primary glaucoma or POAG (Table 4, Fig. 1). Dose lagging (0, 10, 15, 20-year lag periods), inclusion of additional adjustments for non-radiation factors (smoking index, cataract and cataract surgery, smoking status and alcohol consumption) did not significantly modify the observed findings. Exclusion of adjustments for hypertension, BMI and DM did not considerably affect the risk estimate either. Sex, attained age and age at first employment at the enterprise did not significantly modify risk for primary glaucoma (*p* = 0.27, p = 0.38 and p = 0.27, respectively) and for POAG (*p* = 0.28, *p* > 0.5 and *p* = 0.31, respectively).

**Table 3:** RR of primary glaucoma and POAG incidence by dose from external γ-rays, neutron dose and γ + neutron dose.

Cumulative dose (Gy), range	Mean cumulative dose (Gy)	Total primary glaucoma					POAG		
		PYR	Number of cases	RR (95% CI)	*p* value	PYR	Number of cases	RR (95% CI)	*p* value
**Baseline analysis (5 year lag)** ^**a**^ **: RR of primary glaucoma and POAG incidence by brain absorbed dose from external γ-rays**									
0–0.25	0.06	345,381	237	1 (reference)	—	345,512	228	1 (reference)	—
0.25–0.5	0.36	61,697	71	0.87 (0.65–1.15)	0.332	61,699	70	0.88 (0.66–1.17)	0.391
0.5–0.75	0.62	32,775	33	0.76 (0.50–1.10)	0.158	32,794	31	0.73 (0.48–1.07)	0.121
0.75–1.0	0.87	23,771	35	0.90 (0.59–1.32)	>0.50	23,800	35	0.94 (0.62–1.39)	>0.50
1.0–1.25	1.12	16,275	24	0.95 (0.58–1.48)	>0.50	16,275	24	0.99 (0.60–1.56)	>0.50
1.25–1.5	1.37	12,744	19	0.90 (0.51–1.47)	>0.50	12,765	18	0.91 (0.51–1.50)	>0.50
1.5–2.0	1.72	17,407	33	1.22 (0.80–1.81)	0.344	17,409	32	1.22 (0.79–1.82)	0.360
≥2.0	2.57	15,777	24	0.89 (0.54–1.39)	>0.50	15,778	23	0.87 (0.53–1.38)	>0.50
**Sensitivity analysis (5 year lag)** ^**b**^ **: RR of primary glaucoma and POAG incidence by brain absorbed dose from neutrons**									
0–0.001	0.0004	50,538	57	1 (reference)	—	50,554	56	1 (reference)	—
0.001–0.0025	0.0016	17,990	21	0.78 (0.41, 1.42)	0.438	17,990	21	0.83 (0.43, 1.51)	>0.50
0.0025–0.005	0.0034	9,112	22	1.29 (0.65, 2.51)	0.462	9,130	21	1.20 (0.60, 2.36)	>0.50
0.005–0.01	0.0068	2,884	3	0.34 (0.05, 1.23)	0.158	2,884	3	0.34 (0.05, 1.22)	0.157
≥0.01	0.0224	1,178	3	2.06 (0.42, 7.37)	0.310	1,178	3	2.13 (0.43, 7.73)	0.292
**Sensitivity analysis (5 year lag)** ^**a**^ **: RR of primary glaucoma and POAG incidence by the unweighted sum of brain absorbed γ + neutron dose**									
0–0.25	0.06	345,321	237	1 (reference)	—	345,441	228	1 (reference)	—
0.25–0.5	0.36	61,694	71	0.86 (0.65, 1.14)	0.317	61,700	70	0.88 (0.67, 1.17)	0.394
0.5–0.75	0.62	32,727	33	0.74 (0.50, 1.07)	0.119	32,748	31	0.72 (0.48, 1.04)	0.099
0.75–1.0	0.87	23,787	35	0.99 (0.67, 1.41)	>0.50	23,817	35	1.02 (0.69, 1.47)	>0.50
1.0–1.25	1.12	16,343	24	1.02 (0.64, 1.56)	>0.50	16,345	24	1.06 (0.67, 1.61)	>0.50
1.25–1.5	1.37	12,744	19	0.99 (0.59, 1.57)	>0.50	12,765	18	0.97 (0.57, 1.56)	>0.50
1.5–2.0	1.72	17,428	33	1.14 (0.76, 1.67)	>0.50	17,430	32	1.15 (0.75, 1.69)	>0.50
≥2.0	2.57	15,783	24	0.86 (0.53, 1.34)	>0.50	15,786	23	0.84 (0.51, 1.32)	0.476

*Notes:* stratified by sex, attained age, birth cohort, diagnosed with hypertension, body mass index, diagnosed with diabetes mellitus and neutron dose for external exposure association analysis and vice versa. CI, confidence interval estimated using the profile likelihood. PYR, person-years at risk. RR, relative risk. POAG, primary open-angle glaucoma. *p* values are given, assessed via the Wald statistics.

^a^For all workers.

^b^For workers with neutron exposure.

**Table 4:** Primary glaucoma and POAG incidence risk with external γ-dose, neutron dose and γ + neutron dose.

Analysis type	Total primary glaucoma		POAG	
	ERR/Gy (95% CI)	*p* value^a^	ERR/Gy (95% CI)	*p* value^a^
**Incidence risk associated with brain absorbed dose from external γ-rays** ^**i**^				
Baseline analysis^b^, 5 year lag	0.01 (−0.13, 0.19)	>0.50	0.0003 (−0.13, 0.19)	>0.50
**Sensitivity analysis** ^**b**^ **, in which the first x years following the start of radiation work were assigned to a “zero dose” category when lagging doses by x years**				
0 year lag	0.01 (−0.13, 0.19)	>0.50	0.001 (−0.13, 0.19)	>0.50
10 year lag	0.002 (−0.13, 0.19)	>0.50	−0.003 (−0.14, 0.19)	>0.50
15 year lag	−0.001 (−0.13, 0.19)	>0.50	−0.005 (−0.14, 0.18)	>0.50
20 year lag	−0.01 (−0.15, 0.17)	>0.50	−0.02 (−0.15, 0.17)	>0.50
**Sensitivity analysis – exclusion stratification, 5 year lag:**				
By hypertension	−0.01 (−0.13, 0.17)	>0.50	−0.01 (−0.14, 0.17)	>0.50
By BMI	0.01 (−0.12, 0.19)	>0.50	0.01 (−0.12, 0.20)	>0.50
By diabetes mellitus	0.03 (−0.11, 0.22)	>0.50	0.02 (−0.12, 0.21)	>0.50
By neutron dose	0.01 (−0.12, 0.20)	>0.50	0.01 (−0.13, 0.19)	>0.50
**Sensitivity analysis – additional stratification, 5 year lag:**				
By smoking index	0.01 (−0.13, 0.20)	>0.50	−0.001 (−0.14, 0.20)	>0.50
By smoking and alcohol	0.07 (−0.10, 0.32)	0.48	0.06 (−0.11, 0.30)	>0.50
By cataract	−0.04 (−0.16, 0.12)	>0.50	−0.05 (−0.17, 0.11)	0.46
By cataract surgery	0.01 (−0.13, 0.20)	>0.50	0.004 (−0.13, 0.20)	>0.50
**Sensitivity analysis** ^**b**^ **– analysis restricted to workers, 5 year lag:**				
Males	−0.05 (−0.19, 0.16)	>0.50	−0.05 (−0.20, 0.15)	>0.50
Females	0.16 (−0.13, 0.63)	0.38	0.15 (−0.14, 0.63)	0.42
*p* value^c^	0.27		0.28	
**Attained age**				
<50 years	3.24 (−2.41, 61.08)	0.35	1.22 (−1.35, 57.68)	>0.50
50–59 years	−0.13 (n/a, 0.41)	0.47	−0.07 (−0.44, 0.58)	>0.50
60–69 years	−0.03 (−0.21, 0.26)	>0.50	−0.02 (−0.20, 0.28)	>0.50
≥70 years	0.06 (−0.16, 0.41)	>0.50	0.02 (−0.20, 0.37)	>0.50
*p* value^d^	0.38		>0.50	
*p* value^e^	0.49		>0.50	
**Age at first employment**				
<30 years	−0.03 (−0.16, 0.18)	>0.50	−0.03 (−0.17, 0.17)	>0.50
30–40 years	−0.19 (n/a, 0.71)	0.23	−0.18 (n/a, 0.72)	0.25
≥40 years	2.98 (−2.31, 26.99)	0.28	1.81 (−1.55, 17.67)	>0.50
*p* value^f^	0.27		0.31	
**Incidence risk associated with brain absorbed dose from neutrons** ^**j**^				
Sensitivity analysis^g^, 5 year lag	22.83 (−33.20, 150.80)	>0.50	23.64 (−33.77, 154.10)	>0.50
**Incidence risk associated with the unweighted sum of brain absorbed γ + neutron dose** ^**i**^				
Sensitivity analysis^h^, 5 year lag	0.01 (−0.12, 0.19)	>0.50	−0.01 (−0.14, 0.17)	>0.50

*Notes:* BMI, body mass index. DM, diabetes mellitus. ERR, excess relative risk. CI, confidence interval estimated using the profile likelihood. n/a, not available. POAG, primary open-angle glaucoma.

^a^*p* values of improvement in fit over null model (with no trend in dose) are given, assessed via the Wald statistics.

^b^Stratified by sex, attained age, birth cohort, diagnosed with hypertension, BMI, diagnosed with DM and neutron dose.

^c^Tested for heterogeneity between males and females, assessed via the likelihood ratio test.

^d^Tested for heterogeneity between groups of workers of different attained age, assessed via the likelihood ratio test.

^e^Tested for a log-linear trend in the ERR/Gy with attained age, assessed via the likelihood ratio test.

^f^Tested for heterogeneity between groups of workers of different age at first employment, assessed via the likelihood ratio test.

^g^Stratified by sex, attained age, birth cohort, diagnosed with hypertension, BMI, diagnosed with DM and dose from external γ-ray.

^h^Stratified by sex, attained age, birth cohort, diagnosed with hypertension, BMI, diagnosed with DM.

^i^For all workers.

^j^For workers with neutron exposure.

Results of the additional analysis providing odds ratios of various risk factors for primary glaucoma and POAG using the logit regression are summarized in Table S3. The analysis demonstrated that its results are in good agreement with the results of the analyses based on the Poisson regression described above.

## Discussion

This is a retrospective cohort study with a number of advantages, such as the large size of the study cohort, information on both sexes (with 25.4% of females), the extensive follow-up period (up to 70 years), availability of medical information and data on non-radiation risk factors over the entire follow-up period. The main advantage of the present study are the mandatory annual health examinations over the entire follow-up. The annual health examinations were performed using a standard protocol and included a mandatory check by an ophthalmologist and an ophthalmological examination using conventional techniques regardless of workers’ sex, age, working site, occupation, radiation dose, etc.

As expected, the present study found significant associations between primary glaucoma and some known non-radiation risk factors such as sex, attained age, cataract diagnosed before glaucoma manifestation, and cataract removal surgery. These results are in line with those observed in other studies^3,4,23–25^.

The present study did not reveal any significant associations between primary glaucoma incidence and doses from external γ + neutron radiation exposure and its separate components (γ-rays, neutrons) whether adjusted for each other or not. Further one-by-one exclusion of known risk factors (hypertension, BMI, DM) in the model did not modify the ERR/Gy estimate either for primary glaucoma or for POAG (Table 4). While investigating effects following fractionated exposures at dose above tens Gy (each fraction delivered at high dose rate), a number of studies have demonstrated neovascular glaucoma as one of the normal tissue complications following ocular or head and neck radiotherapy, which have been mediated by plaque formation in microcirculation and further neovascularization^5–11^. As shown in Table 5, following single, acute exposure at several Gy, glaucoma risk has been reported in Japanese atomic bomb survivors: Yamada *et al*. first reported that incidence of glaucoma in aggregate (i.e., without distinguishing any type of glaucoma) significantly decreases with increasing radiation dose^26^. In a subsequent study, and as shown in Table 5, Kiuchi *et al*. reported an Odds ratio at 1 Gy of 1.31 (95% CIs: 1.11, 1.53, *p* = 0.001) for normal-tension glaucoma, albeit with no significant associations between radiation doses and other types of glaucoma^12^, and the authors in their recent paper^13^ confirmed the significant odds ratio at 1 Gy for NTG for both right and left eyes (Table 5). However, Kiuchi *et al*.^12^ note that it was a screening study and the cohort member participation rate in that study was relatively low, underlining a need to interpret these findings with caution. Following occupational protracted exposures at low dose, there was no literature available until Little *et al*. very recently reported an insignificantly decreased risk for self-reported glaucoma in aggregate in the US radiologic technologists (USRT) cohort^14^ (and see also Table 5), which was based solely on questionnaire information obtained from medically literate workers, without any ophthalmological validation. Accordingly, it remains unclear whether members of the USRT cohort and Mayak workers exhibit a significantly increased risk for normal-tension glaucoma as reported in atomic bomb survivors. As such, our future studies include evaluation of the POAG risk separately for normal-tension type and high-tension type based on the measured IOP data.

**Table 5:** Glaucoma incidence risk following various exposure scenarios.

Reference, year	Study design	Cohort	Follow-up period	Radiation dose (range)	Number of participants	Number of cases	Findings	Adjustments
Kiuchi *et al*.^12^	Screening with the succeeding ophthalmological examination	AHS	2006–2008 (51–53 years after exposure)	Eye dose 0.57 Gy (0–4.14)	1589	Normal-tension POAG: 226	OR at 1 Gy = 1.31 (95% CIs: 1.11, 1.53, *p* = 0.001)	Sex, age, city, cataract surgery, diabetes mellitus
						POAG with IOP > 21 mmHg: 36	OR at 1 Gy = 0.79 (95% CIs: 0.52, 1.21, *p* = 0.28)	
						PACG: 25	OR at 1 Gy = 0.54 (95% CIs: 0.29, 1.02, *p* = 0.06)	
Kiuchi *et al*.^13^	Screening with the succeeding ophthalmological examination	AHS	2006–2008 (51–53 years after exposure)	Mean radiation dose 0.66 Gy (NTG subjects)	1640	NTG: 153	OR at 1 Gy = 1.39 (95% CI: 1.15, 1.69, *p* < 0.01)	Sex. Age, city, smoking, hypertension, dyslipidemia, diabetes, BMI, CRP
						POAG: 25	OR at 1 Gy = 0.79 (95% CI: 0.32, 1.82, *p* = 0.54)	
						PACG: 18	OR at 1 Gy = 0.44 (95% CI: 0.13, 1.54, *p* = 0.20)	
Little *et al*.^14^	Questionnaire-based screening (self-reported diagnosis)	USRT	1983–1989 1994–1998 2003–2005 2012–2014 (13.2 years of a mean follow-up per person)	Eye lens dose 0.058 Gy (0.024–0.071)	69,568	1631	ERR/Gy = −0.57 (95% CIs: −1.46, 0.60, *p* = 0.304)	Sex, ethnicity, year of birth, diabetes mellitus, BMI, smoking status
Azizova *et al*., 2019 (present study)	Cohort study based on mandatory annual ophthalmological examinations throughout the whole follow-up period	Mayak PA	1948–2008 (35.7 years of a mean follow-up per person)	Brain absorbed dose from external γ-rays 0.44 Gy (0–8.4)	22,377	Primary glaucoma: 476	ERR/Gy = 0.01 (95% CIs: −0.13, 0.19, *p* > 0.50)	Sex, attained age, birth cohort, hypertension, BMI, DM and neutron dose
						POAG: 461	ERR/Gy = 0.0003 (95% CIs: −0.13, 0.19, *p* > 0.50)	

AHS, Adult Health Study. BMI, body mass index, CRP, C-reactive protein. DM, diabetes mellitus. ERR, excess relative risk. IOP, intraocular pressure. OR, odds ratio. PA, Production Association. PACG, primary angle-closure glaucoma. POAG, primary open-angle glaucoma. USRT, US Radiologic Technologists.

Biological mechanisms underlying radiation-induced glaucoma remain unclear^27^. The most likely mechanisms that have been suggested thus far involve impaired retinal circulation since some data have demonstrated the impaired circulation at 30–45 years after radiation exposure^28^ as well as retinal vessel atherosclerosis^29^. In addition, other possible mechanisms of radiation glaucoma may include neural excitotoxicity^30^, inflammation^31^, genetic predisposition^32–35^, autoimmune reactions^36^ and retinal circulation disorders^37,38^. Some investigators believe that the most likely of them might be retinal circulation disorders^12^, since there is evidence of radiation-induced blood flow decrease in the retina 30 or 45 years after the exposure^28^, as well as of the significant association of retinal arteriosclerosis with radiation exposure in atomic bomb survivors 55 years after the exposure^29^.

In conclusion, significant association was found neither between cumulative dose from external γ-rays nor cumulative neutron dose nor the unweighted sum of cumulative γ and neutron doses and incidence of primary glaucoma and POAG in the cohort of Mayak PA workers. Glaucoma incidence was significantly associated with non-radiation risk factors (sex, attained age, and cataract diagnosed prior to glaucoma). Further analyses to distinguish normal-tension type and high-tension type are needed.

## Supplementary information

Study dataset details and Statistical analysis descroption

## Data Availability

The dataset is the intellectual property of the Southern Urals Biophysics Institute, Ozyorsk, Chelyabinsk Region, 456780, Russia. For privacy reasons it is not publicly available. Any access to the Mayak Workers Cohort must be approved by SUBI’s Institutional Review Board. These restrictions on data availability are imposed by Federal Act No. 323 of 21 November 2011 on the basics of health care for Russian citizens and Federal Act No. 152 of 27 July 2014 on personal data. To request the data used in the presented analyses, please, contact Dr. Tamara Azizova, the head of the clinical department of the Southern Urals Biophysics Institute. Any access to the Mayak Workers Cohort must be approved by SUBI’s Institutional Review Board. Please, contact Dr. Valentina Rybkina, MD, leading researcher of SUBI, IAB member (rybkina@subi.su; +73513029953).

## References

[1] Thylefors B, Negrel AD (1994). The global impact of glaucoma. Bull World Health Organ..

[2] Quigley HA, Broman AT (2006). Number of people with glaucoma worldwide in 2010 and 2020. Br J Ohthalmol..

[3] Le A, Mukesh BN, McCarty CA, Taylor HR (2003). Risk factors associated with the incidence of open-angle glaucoma: the visual impairment project. Invest Ophthalmol Vis Sci..

[4] Leske MC, Wu SY, Hennis A, Honkanen R, Nemesure B (2008). BESs Study Group. Risk factors for incident open-angle glaucoma: the Barbados Eye Studies. Ophthalmology..

[5] Shields CL (2001). Plaque radiotherapy for retinoblastoma: long-term tumor control and treatment complications in 208 tumors. Ophthalmology..

[6] Shields CL (2002). Combined plaque radiotherapy and transpupillary thermotherapy for choroidal melanoma: tumor control and treatment complications in 270 consecutive patients. Arch Ophthalmol..

[7] Dieckmann K (2003). LINAC based stereotactic radiotherapy of uveal melanoma: 4 years clinical experience. Radiother Oncol..

[8] Takeda A (1999). Late retinal complications of radiation therapy for nasal and paranasal malignancies: relationship between irradiated-dose area and severity. Int J Radiat Oncol Biol Phys..

[9] Bechrakis N (2002). ^125^I Brachytherapy vs. trans-scleral tumor resection. Ophthalmology..

[10] Desjardins L (2012). Current concepts in uveal melanoma. Dev. Ophthalmol..

[11] Hayashi K (2018). Efficacy and safety of carbon-ion radiotherapy for lacrimal gland carcinomas with extraorbital extension: a retrospective cohort study. Oncotarget..

[12] Kuichi Y (2013). Glaucoma in atomic bomb survivors. Radiat. Res..

[13] Kuichi Y (2019). Association between radiation, glaucoma subtype, and retinal vessel diameter in atomic bomb survivors. Sci. Rep..

[14] Little MP (2018). Occupational radiation exposure and glaucoma and macular degeneration in the US radiologic technologists. Sci Rep..

[15] ICD-9 guidelines for coding diseases, injuries and causes of death/revision 1975. (Geneva, Switzerland: WHO, 1980).

[16] Azizova TV (2008). The “Clinic” medical-dosimetric database of Mayak production association workers: structure, characteristics and prospects of utilization. Health Phys..

[17] Khokhryakov VV (2013). Mayak Worker Dosimetry System 2008 (MWDS-2008): Assessment of internal alpha-dose from measurement results of plutonium activity in urine. Health Phys..

[18] ICRP (2007). 2007 Recommendations of the International Commission on Radiological Protection. ICRP Publication 103. Ann. ICRP.

[19] Azizova TV, Bragin EV, Hamada N, Bannikova MV (2016). Risk of Cataract Incidence in a Cohort of Mayak PA Workers following Chronic Occupational Radiation exposure. PLoS ONE.

[20] Azizova TV, Hamada N, Grigoryeva ES, Bragin EV (2018). Risk of Various Types of Cataracts in a Cohort of Mayak Workers following Chronic Occupational Exposure to Ionizing Radiation. Eur J Epidemiol.

[21] Azizova TV, Hamada N, Bragib EV, Bannikova MV, Grigoryeva ES (2019). Risk of Cataract Removal Surgery in Mayak PA Workers Occupationally Exposed to Ionizing Radiation over Prolonged Periods. Radiat Environ Biophys.

[22] Preston, D., Lubin, J., Pierce, D. & McConney, M. Epicure Users Guide. (Hirosoft, Seattle, W. A., 1993).

[23] Prokofyeva E, Zrenner E (2012). Epidemiology of Eye Diseases Leading to Blindness. Ophthalmic Res..

[24] Lee JY (2017). Relationship between anthropometric parameters and open angle glaucoma: The Korea National Health and Nutrition Examination Survey. Plos One..

[25] Zhao D, Cho J, Kim MH, Friedman DS, Guallar E (2015). Diabetes, fasting glucose, and the risk of glaucoma: a meta-analysis. Ophthalmology.

[26] Yamada M, Wong FL, Fujiwara S, Akahoshi M, Suzuki G (2004). Noncancer disease incidence in atomic bomb survivors, 1958–1998. Radiat. Res..

[27] Hamada N (2019). Glaucomagenesis following radiation exposure. Mutat Res..

[28] Peiretti E, Slakter JS, Wu S, Iranmanesh R, Yannuzzi LA (2006). Late effect of external eye irradiation on choroidal circulation. Eur J Ophthalmol..

[29] Minamoto A (2004). Cataract in atomic bomb survivors. Int J Radiat Biol..

[30] Caprioli J (2007). Glaucoma is a neuronal disease. Eye..

[31] Leibovitch I (2005). C-reactive protein levels in normal tension glaucoma. J Glaucoma..

[32] Shibuya E (2008). Association of Toll-like receptor 4 gene polymorphisms with normal tension glaucoma. Invest Ophthalmol Vis Sci..

[33] Wang CY (2007). Investigation of the association between interleukin-1beta polymorphism and normal tension glaucoma. Mol Vis..

[34] Jeoung JW (2007). Investigation of the association between normal-tension glaucoma and single nucleotide polymorphisms in natriuretic peptide gene. Korean J Ophthalmol.

[35] Mabuchi F (2007). The OPA1 gene polymorphism is associated with normal tension and high tension glaucoma. Am J Ophthalmol..

[36] Hammam T, Montgomery D, Morris D, Imrie F (2008). Prevalence of serum autoantibodies and paraproteins in patients with glaucoma. Eye..

[37] Flammer J (2002). The impact of ocular blood flow in glaucoma. Prog Retin Eye Res..

[38] Amerasinghe N (2008). Evidence of retinal vascular narrowing in glaucomatous eyes in an Asian population. Invest Ophthalmol Vis Sci..

