# Supplementary material for: Glaucoma incidence risk in a cohort of Mayak PA workers occupationally exposed to ionizing radiation
Source: Sci Rep. 2019 Aug 28;9:12469. doi: 10.1038/s41598-019-48915-6 (PMC6713741; doi:10.1038/s41598-019-48915-6)
Supplement: Supplementary file 1 — Study dataset details and Statistical analysis descroption [file 41598_2019_48915_MOESM1_ESM.pdf]

Glaucoma incidence risk in a cohort of Mayak PA workers occupationally exposed to ionizing radiation

Evgeny V. Bragin[1], Tamara V. Azizova[1], Maria V. Bannikova[1], Evgeniya S. Grigoryeva[1] & Nobuyuki Hamada[2]

[1]Southern Urals Biophysics Institute (SUBI), Ozyorskoe Shosse 19, Ozyorsk Chelyabinsk Region, 456780, Russia.

[2]Radiation Safety Research Center, Nuclear Technology Research Laboratory, Central Research Institute of Electric Power Industry (CRIEPI), 2-11-1 Iwado-kita, Komae, Tokyo 201-8511, Japan. Correspondence and requests for materials should be addressed to T.V.A. (email: clinic@subi.su)

Supplementary Table S1. Study cohort characteristics.

Distribution of workers by age at first employment at the facility						
Age at first employment, years	Males		Females		Both sexes	
	Number	%	Number	%	Number	%
<20	5399	32.36	771	13.54	6170	27.58
20–24	5863	35.13	2000	35.16	7863	35.14
25–29	2607	15.62	1108	19.48	3715	16.60
30–34	1103	6.61	632	11.11	1735	7.75
35–39	816	4.89	608	10.69	1424	6.36
≥40	900	5.39	570	10.02	1470	6.57
Total	16688	100.00	5689	100.00	22377	100.00
Distribution of workers by period of first employment						
Period of first employment	Males		Females		Both sexes	
	Number	%	Number	%	Number	%
1948–1953	5485	32.86	2921	51.34	8406	37.56
1954–1958	3233	19.37	658	11.57	3891	17.38
1959–1963	3221	19.30	616	10.83	3837	17.15
1964–1968	1370	8.21	348	6.12	1718	7.68
1969–1972	827	4.96	224	3.94	1051	4.70
1973–1978	1770	10.61	601	10.56	2371	10.60
1979–1982	782	4.69	321	5.64	1103	4.93
Total	16688	100.00	5689	100.00	22377	100.00
Distribution of workers by age as of the date of primary glaucoma diagnosis						
Age at diagnosis, years	Males		Females		Both sexes	
	Number	%	Number	%	Number	%
<50	17	5.40	7	4.35	24	5.04
50–59	68	21.59	11	6.83	79	16.60
60–69	148	46.98	63	39.13	211	44.33
70–79	73	23.17	68	42.24	141	29.62
≥80	9	2.86	12	7.45	21	4.41
Total	315	100.00	161	100.00	476	100.00
Distribution of workers by age as of the date of primary open-angle glaucoma diagnosis						
Age at diagnosis, years	Males		Females		Both sexes	
	Number	%	Number	%	Number	%
<50	16	5.21	5	3.25	21	4.55
50–59	67	21.82	9	5.84	76	16.49
60–69	145	47.23	63	40.91	208	45.12
70–79	71	23.13	64	41.56	135	29.28
≥80	8	2.61	13	8.44	21	4.56
Total	307	100.00	154	100.00	461	100.00
Distribution of workers by age at the end of the follow-up						
Age at the end of the follow-up, years	Males		Females		Both sexes	
	Number	%	Number	%	Number	%
<40	6761	40.51	1694	29.78	8455	37.78
40–49	1767	10.59	370	6.50	2137	9.55
50–59	3016	18.07	714	12.55	3730	16.67
60–69	2735	16.39	915	16.08	3650	16.31
≥70	2409	14.44	1996	35.09	4405	19.69
Total	16688	100.00	5689	100.00	22377	100.00
Distribution of workers by duration of employment at one of the main facilities						
Employment duration, years	Males		Females		Both sexes	
	Number	%	Number	%	Number	%
<1	839	5.03	217	3.81	1056	4.72
1–10	6149	36.85	2012	35.37	8161	36.47

≥10	9700	58.12	3460	60.82	13160	58.81
Total	16688	100.00	5689	100.00	22377	100.00
Distribution of workers by cumulative brain absorbed dose from external γ-rays						
Cumulative external γ-dose, Gy	Males		Females		Both sexes	
	Number	%	Number	%	Number	%
<0.25	9518	57.03	3646	64.09	13164	58.83
0.25–0.5	2522	15.12	672	11.81	3194	14.27
0.5–1.0	2120	12.70	713	12.53	2833	12.66
≥1.0	2528	15.15	658	11.57	3186	14.24
Total	16688	100.00	5689	100.00	22377	100.00
Distribution of workers by cumulative brain absorbed dose from neutrons						
Cumulative neutron dose, Gy	Males		Females		Both sexes	
	Number	%	Number	%	Number	%
Unmeasured 0.0	13213	79.18	5081	89.32	18294	81.75
<0.001	2110	12.64	431	7.57	2541	11.36
0.001–0.005	1173	7.03	140	2.46	1313	5.87
≥0.005	192	1.15	37	0.65	229	1.02
Total	16688	100.00	5689	100.00	22377	100.00
Distribution of workers by the unweighted sum of cumulative brain absorbed γ + neutron doses						
Cumulative γ + neutron dose, Gy	Males		Females		Both sexes	
	Number	%	Number	%	Number	%
<0.25	9517	57.03	3646	64.09	13163	58.82
0.25–0.5	2521	15.11	672	11.81	3193	14.27
0.5–1.0	2116	12.68	713	12.53	2829	12.64
≥1.0	2534	15.18	658	11.57	3192	14.27
Total	16688	100.00	5689	100.00	22377	100.00

Supplementary Table S2. Risk analysis results for primary glaucoma and POAG incidence in relation to non-radiation factors.

Factors		Total primary glaucoma			POAG		
		RR (95% CI)	Number of cases	*p* value	RR (95% CI)	Number of cases	*p* value
RR for various calendar periods of glaucoma diagnosis date (compared to 2006–2008)							
Males	1948–1955	–	0	–	–	0	–
	1956–1965	–	0	–	–	0	–
	1966–1975	**0.20 (0.04, 0.92)**	4	**0.046**	**0.13 (0.02, 0.67)**	3	**0.021**
	1976–1985	0.57 (0.22, 1.39)	32	0.221	0.48 (0.19, 1.21)	31	0.128
	1986–1995	**0.52 (0.30, 0.91)**	83	**0.022**	**0.47 (0.26, 0.82)**	78	**0.008**
	1996–2005	**0.64 (0.43, 0.96)**	143	**0.031**	**0.63 (0.42, 0.95)**	143	**0.027**
	2006–2008	1 (reference)	53	–	1 (reference)	52	–
Females	1948–1955	–	0	–	–	0	–
	1956–1965	–	0	–	–	0	–
	1966–1975	0.82 (0.09, 6.64)	7	>0.50	1.62 (0.15, 18.41)	6	>0.50
	1976–1985	0.95 (0.26, 3.41)	17	>0.50	1.37 (0.36, 5.32)	13	>0.50
	1986–1995	**0.44 (0.19, 0.99)**	30	**0.049**	0.50 (0.22, 1.14)	31	0.097
	1996–2005	0.71 (0.41, 1.27)	79	0.233	0.76 (0.43, 1.38)	78	0.350
	2006–2008	1 (reference)	28	–	1 (reference)	26	–
RR for workers first employed at one of the main facilities in 1954 or later compared to those before 1954							
Males	1948–1953	1 (reference)	115	–	1 (reference)	109	–
	1954–1958	0.98 (0.71, 1.36)	78	>0.50	1.04 (0.74, 1.44)	78	>0.50
	1959–1963	0.78 (0.54, 1.12)	61	0.182	0.81 (0.56, 1.16)	60	0.262
	1964–1968	0.78 (0.47, 1.24)	26	0.303	0.79 (0.47, 1.27)	25	0.347
	1969–1972	0.65 (0.29, 1.28)	9	0.248	0.69 (0.31, 1.36)	9	0.320
	1973–1978	1.09 (0.60, 1.88)	22	>0.50	1.15 (0.63, 2.00)	22	>0.50
	1979–1982	0.62 (0.18, 1.59)	4	0.374	0.65 (0.19, 1.69)	4	0.434
Females	1948–1953	1 (reference)	76	–	1 (reference)	73	–
	1954–1958	1.23 (0.77, 1.91)	26	0.365	1.28 (0.80, 1.98)	26	0.294
	1959–1963	0.97 (0.60, 1.52)	26	>0.50	0.98 (0.60, 1.54)	25	>0.50
	1964–1968	1.00 (0.49, 1.86)	11	>0.50	0.93 (0.44, 1.77)	10	>0.50
	1969–1972	**0.30 (0.05, 0.97)**	2	**0.049**	**0.31 (0.05, 0.99)**	2	**0.049**
	1973–1978	1.32 (0.68, 2.42)	16	0.398	1.17 (0.58, 2.21)	14	>0.50
	1979–1982	0.99 (0.28, 2.72)	4	>0.50	1.00 (0.28, 2.78)	4	>0.50
RR for various groups of workers by age at first employment at the facility (compared to <20 years old group)							
Males	<20	1 (reference)	84	–	1 (reference)	83	–
	20–25	0.81 (0.59, 1.11)	110	0.185	0.81 (0.59, 1.11)	108	0.187
	25–30	**0.68 (0.46, 0.99)**	49	**0.049**	**0.65 (0.43, 0.96)**	46	**0.033**
	30–35	0.68 (0.42, 1.09)	29	0.116	0.67 (0.40, 1.07)	28	0.103
	35–40	0.60 (0.33, 1.03)	20	0.074	0.61 (0.34, 1.05)	20	0.086
	≥40	0.84 (0.47, 1.44)	23	>0.50	0.85 (0.47, 1.46)	22	>0.50
Females	<20	1 (reference)	16	–	1 (reference)	15	–
	20–25	1.21 (0.68, 2.25)	50	0.370	1.28 (0.71, 2.43)	50	0.425
	25–30	1.12 (0.58, 2.20)	28	0.112	1.13 (0.58, 2.28)	27	>0.50
	30–35	1.13 (0.56, 2.28)	21	0.207	1.13 (0.56, 2.34)	20	>0.50
	35–40	1.04 (0.53, 2.08)	22	0.111	1.05 (0.52, 2.14)	21	>0.50
	≥40	1.04 (0.51, 2.14)	24	0.433	0.94 (0.44, 2.00)	21	>0.50
RR for ever-smokers vs. never-smokers							
Males	Never-smoker	1 (reference)	75	–	1 (reference)	74	–
	Ever-smokers	0.94 (0.73, 1.23)	239	>0.50	0.93 (0.72, 1.22)	232	>0.50
	Unknown	0.46 (0.03, 2.08)	1	0.441	0.48 (0.03, 2.16)	1	0.463
Females	Never-smoker	1 (reference)	152	–	1 (reference)	146	–
	Ever-smokers	1.08 (0.46, 2.15)	7	>0.50	1.13 (0.48, 2.24)	7	>0.50
	Unknown	0.56 (0.09, 1.78)	2	0.420	0.29 (0.02, 1.31)	1	0.219
RR for workers with smoking index above 0 compared to non-smokers							
Males	0 (non-smokers)	1 (reference)	75	–	1 (reference)	74	–
	Unknown	0.80 (0.51, 1.22)	28	0.312	0.79 (0.50, 1.21)	27	0.285
	<10	0.95 (0.59, 1.46)	26	>0.50	0.96 (0.60, 1.48)	26	>0.50
	10–20	1.08 (0.72, 1.59)	38	>0.50	1.04 (0.69, 1.54)	36	>0.50
	≥20	0.94 (0.71, 1.25)	148	>0.50	0.93 (0.70, 1.24)	144	>0.50
Females	0 (non-smokers)	1 (reference)	152	–	1 (reference)	146	–
	Unknown	0.61 (0.15, 1.62)	3	0.397	0.42 (0.07, 1.33)	2	0.228
	<10	1.94 (0.59, 4.61)	4	0.194	2.06 (0.63, 4.90)	4	0.157
	10–20	0.95 (0.05, 4.27)	1	>0.50	0.99 (0.06, 4.46)	1	>0.50
	≥20	0.50 (0.03, 2.22)	1	0.485	0.51 (0.03, 2.27)	1	0.499

RR for workers drinking alcohol compared to never-drinkers						
Males	Never-drinkers	1 (reference)	10	–	1 (reference)	9	–
	Ever-drinkers	0.70 (0.39, 1.42)	294	0.272	0.77 (0.42, 1.61)	287	0.432
	Unknown	0.68 (0.29, 1.64)	11	0.380	0.76 (0.32, 1.90)	11	>0.50
Females	Never-drinkers	1 (reference)	90	–	1 (reference)	87	–
	Ever-drinkers	1.11 (0.80, 1.54)	68	>0.50	1.10 (0.80, 1.53)	65	>0.50
	Unknown	0.42 (0.10, 1.12)	3	0.138	**0.29 (0.05, 0.92)**	2	**0.049**
RR for workers with vs. without cataract removal surgery							
Males	No surgery	1 (reference)	296	–	1 (reference)	289	–
	After surgery	1.57 (0.94, 2.47)	19	0.069	1.53 (0.90, 2.43)	18	0.093
Females	No surgery	1 (reference)	152	–	1 (reference)	145	–
	After surgery	1.29 (0.60, 2.44)	9	0.465	1.33 (0.62, 2.52)	9	0.415

Notes: Bold font, $p < 0.05$. CI, confidence interval estimated using the profile likelihood. POAG, primary open-angle glaucoma. RR, relative risk. p values are given, assessed via the Wald statistics.

Supplementary statistical analysis

Hypertension was assigned when both systolic blood pressure was >140 mmHg and diastolic blood pressure was >90 mmHg. Hypertension was taken into account as a qualitative parameter categorized as ‘unknown’, ‘hypertension-free’ and ‘hypertension’. BMI was estimated as body weight (kg) divided by squared height (m^2), and was taken into account as a qualitative parameter categorized as ‘unknown’, ‘below normal’, ‘normal’, ‘above normal’ and ‘obese’. BMI <18.5 kg m^{-2} was referred as body weight deficit; BMI 18.5–24.9 kg m^{-2} was referred as a normal body weight; BMI 25.0–29.9 kg m^{-2} was referred as an excessive body weight; and BMI ≥30 kg m^{-2} was referred as obesity. In the present study, we took into account BMI and hypertension registered at the first pre-employment medical health examination (before a person started working at the Mayak PA).

Data on smoking habits were taken into account over the entire follow-up period and estimated with qualitative and quantitative indices. The qualitative index included values ‘unknown’, ‘never smoker’, and ‘ever smoker’. ‘Never smoker’ was assumed to be a worker who reported to have never smoked during a series of annual mandatory medical examinations. The quantitative index (referred to as the smoking index) was calculated as the mean number of cigarette packs smoked in a day times years of smoking. The smoking index was measured by pack-years, and for ‘never-smokers’ was equated to zero.

Data on alcohol consumption were taken into account over the entire follow-up period and estimated with a qualitative parameter with values ‘unknown’, ‘ever drinker’, and ‘never drinker’. ‘Never drinker’ was assumed to be a worker who reported to have never drunk alcohol during a series of annual mandatory medical examinations.

Papers reporting glaucoma risks following chronic radiation exposure are very sparse. In our work, we compare findings observed in four studies (Table 5). All these studies used different analysis techniques. Kiuchi et al [1, 2] used generalized estimating equations (GEE) to find the association between glaucoma eye-specific prevalence and explanatory variables and assumed logistic models for eye-specific prevalence. Little et al [3] used Cox proportional hazards models for estimating risks for glaucoma and macular degeneration. Azizova et al (present study) used the Poisson regression for estimating excess relative risk per unit dose (ERR/Gy). Kiuchi et al. reported significant Odds ratio at 1 Gy for normal-tension glaucoma [1, 2]. Little et al [3] and Azizova et al (present study) did not find significant associations between radiation doses and glaucoma.

To avoid risk underestimation due to the selected analysis technique we performed an additional analysis using a logit regression. To perform this analysis the same set of individual data for the study cohort workers as for the Poisson regression based analysis (its results are described in the main paper) was used. The data were compiled as an event-count table using

DATAB module of EPICURE software [4]. All explanatory variables were taken into account at a date when a worker had exited the study, i.e. at the earliest date among the following: a date of the disease diagnosis, a date of death, 31 December 2008 for alive workers still residing in Ozyorsk, date of 'the last medical information' for workers-residents with an unknown vital status and for those who had left Ozyorsk). A unit of the analysis was a person rather than eyes. For both primary glaucoma and POAG the following models were used:

$$\ln\left(\frac{p}{1-p}\right) = \alpha_0 + \alpha_1 S + \alpha_2 SHyp + \alpha_3 SBMI + \alpha_4 SDm + \alpha_5 Age + \alpha_6 SBy + \beta D_{\gamma} \quad \text{Model 1}$$

$$\ln\left(\frac{p}{1-p}\right) = \alpha_0 + \alpha_1 S + \alpha_2 SHyp + \alpha_3 SBMI + \alpha_4 SDm + \alpha_5 Age + \alpha_6 SBy + \beta D_{neutron} \quad \text{Model 2}$$

$$\ln\left(\frac{p}{1-p}\right) = \alpha_0 + \alpha_1 S + \alpha_2 SHyp + \alpha_3 SBMI + \alpha_4 SDm + \alpha_5 Age + \alpha_6 SBy + \beta D_{\gamma+neutron} \quad \text{Model 3}$$

where $Age = (age\ at\ follow\ up\ date - 65)/5$; $By = (birth\ year - 1935)/5$; S is an indicator of sex, *Hyp* is an indicator of hypertension, *BMI* is an indicator of body mass index, *Dm* is an indicator of diabetes mellitus, and α and β are regression parameters. Common odds ratio for a radiation exposure of 1 Gy is *exp(β),* where β is an estimate of the dose-response parameter.

The GMBO module of the EPICURE software was used to run the analyses. Two-sided P values and 95% confidence intervals were based on the Wald statistics.

The results of the analysis are summarized in Table S3.

The analysis demonstrated that the results based on the logit regression are in good agreement with the results based on the Poisson regression.

Table S3. Odds Ratio of Various Risk Factors for Primary glaucoma and POAG.

Analysis type	**Total primary glaucoma**		**POAG**	
	Odds ratio (95% CI)	*p* value[1]	Odds ratio (95% CI)	*p* value[1]
**Model 1[2]**				
Brain absorbed dose from external γ-rays at 1 Gy	1.04 (0.88, 1.19)	> 0.50	1.04 (0.89, 1.18)	> 0.50
Sex (females vs. males)	0.90 (0.17, 1.64)	> 0.50	0.89 (0.17, 1.61)	> 0.50
Males, age at follow-up date (given per 5 years at reference age 65)	1.06 (1.02, 1.10)	0.003	1.06 (1.02, 1.10)	0.003
Females, age at follow-up date (given per 5 years at reference age 65)	1.06 (1.00, 1.11)	0.036	1.06 (1.00, 1.11)	0.033
Males, birth year (given per 5 years at reference year 1935)	1.00 (0.96, 1.04)	> 0.50	1.00 (0.96, 1.04)	> 0.50
Females, birth year (given per 5 years at reference year 1935)	0.95 (0.87, 1.04)	0.261	0.96 (0.87, 1.04)	0.302
Males, hypertension (yes vs. no)	0.96 (0.70, 1.22)	> 0.50	0.95 (0.70, 1.20)	> 0.50
Females, hypertension (yes vs. no)	0.90 (0.39, 1.42)	> 0.50	0.91 (0.41, 1.42)	> 0.50
Males, BMI (<18.5 kg m^{-2} vs. 18.5–24.9 kg m^{-2})	0.91 (0.17, 1.65)	> 0.50	0.91 (0.17, 1.65)	> 0.50
Males, BMI (25–29.9 kg m^{-2} vs. 18.5–24.9 kg m^{-2})	0.92 (0.65, 1.20)	> 0.50	0.92 (0.64, 1.19)	> 0.50
Males, BMI (≥30 kg m^{-2} vs. 18.5–24.9 kg m^{-2})	1.07 (0.02, 2.12)	> 0.50	1.08 (0.03, 2.13)	> 0.50
Females, BMI (<18.5 kg m^{-2} vs. 18.5–24.9 kg m^{-2})	0.71 (-0.46, 1.88)	> 0.50	0.71 (-0.46, 1.87)	> 0.50
Females, BMI (25–29.9 kg m^{-2} vs. 18.5–24.9 kg m^{-2})	1.06 (0.63, 1.48)	> 0.50	1.02 (0.61, 1.44)	> 0.50
Females, BMI (≥30 kg m^{-2} vs. 18.5–24.9 kg m^{-2})	1.13 (0.31, 1.94)	> 0.50	1.09 (0.30, 1.88)	> 0.50
Males, diabetes mellitus (yes vs. no)	1.09 (0.56, 1.63)	> 0.50	1.08 (0.55, 1.61)	> 0.50
Females, diabetes mellitus (yes vs. no)	1.17 (0.54, 1.79)	> 0.50	1.17 (0.56, 1.79)	> 0.50
**Model 2[3]**				
Brain absorbed dose from neutrons at 1 Gy	6.04 (-39.42, 51.51)	> 0.50	6.33 (-39.04, 51.70)	> 0.50
Sex (females vs. males)	0.90 (0.19, 1.60)	> 0.50	0.89 (0.19, 1.58)	> 0.50
Males, age at follow-up date (given per 5 years at reference age 65)	1.06 (1.02, 1.10)	0.002	1.06 (1.02, 1.10)	0.002
Females, age at follow-up date (given per 5 years at reference age 65)	1.06 (1.01, 1.11)	0.029	1.06 (1.01, 1.11)	0.026
Males, birth year (given per 5 years at reference year 1935)	0.99 (0.96, 1.03)	> 0.50	1.00 (0.96, 1.03)	> 0.50
Females, birth year (given per 5 years at reference year 1935)	0.95 (0.87, 1.03)	0.222	0.95 (0.87, 1.03)	0.261
Males, hypertension (yes vs. no)	0.96 (0.72, 1.21)	> 0.50	0.95 (0.71, 1.20)	> 0.50
Females, hypertension (yes vs. no)	0.90 (0.41, 1.40)	> 0.50	0.91 (0.43, 1.40)	> 0.50
Males, BMI (<18.5 kg m^{-2} vs. 18.5–24.9 kg m^{-2})	0.91 (0.20, 1.62)	> 0.50	0.91 (0.21, 1.62)	> 0.50
Males, BMI (25–29.9 kg m^{-2} vs. 18.5–24.9 kg m^{-2})	0.92 (0.66, 1.19)	> 0.50	0.92 (0.65, 1.18)	> 0.50
Males, BMI (≥30 kg m^{-2} vs. 18.5–24.9 kg m^{-2})	1.06 (0.05, 2.07)	> 0.50	1.07 (0.06, 2.07)	> 0.50
Females, BMI (<18.5 kg m^{-2} vs. 18.5–24.9 kg m^{-2})	0.71 (-0.41, 1.82)	> 0.50	0.70 (-0.41, 1.81)	> 0.50
Females, BMI (25–29.9 kg m^{-2} vs. 18.5–24.9 kg m^{-2})	1.05 (0.65, 1.46)	> 0.50	1.02 (0.62, 1.42)	> 0.50

Females, BMI (≥30 kg m^{-2} vs. 18.5–24.9 kg m^{-2})	1.12 (0.34, 1.90)	> 0.50	1.08 (0.32, 1.84)	> 0.50
Males, diabetes mellitus (yes vs. no)	1.09 (0.58, 1.61)	> 0.50	1.08 (0.57, 1.59)	> 0.50
Females, diabetes mellitus (yes vs. no)	1.16 (0.56, 1.76)	> 0.50	1.17 (0.58, 1.76)	> 0.50
**Model 3**[2]				
Unweighted sum of brain absorbed γ + neutron dose at 1 Gy	1.03 (0.88, 1.18)	> 0.50	1.03 (0.89, 1.18)	> 0.50
Sex (females vs. males)	0.90 (0.17, 1.63)	> 0.50	0.89 (0.17, 1.60)	> 0.50
Males, age at follow-up date (given per 5 years at reference age 65)	1.06 (1.02, 1.10)	0.002	1.06 (1.02, 1.10)	0.003
Females, age at follow-up date (given per 5 years at reference age 65)	1.06 (1.00, 1.11)	0.035	1.06 (1.00, 1.11)	0.032
Males, birth year (given per 5 years at reference year 1935)	1.00 (0.96, 1.04)	> 0.50	1.00 (0.96, 1.04)	> 0.50
Females, birth year (given per 5 years at reference year 1935)	0.95 (0.87, 1.04)	0.268	0.96 (0.87, 1.04)	0.312
Males, hypertension (yes vs. no)	0.96 (0.70, 1.21)	> 0.50	0.95 (0.70, 1.20)	> 0.50
Females, hypertension (yes vs. no)	0.90 (0.39, 1.41)	> 0.50	0.91 (0.41, 1.41)	> 0.50
Males, BMI (<18.5 kg m^{-2} vs. 18.5–24.9 kg m^{-2})	0.90 (0.17, 1.63)	> 0.50	0.90 (0.17, 1.63)	> 0.50
Males, BMI (25–29.9 kg m^{-2} vs. 18.5–24.9 kg m^{-2})	0.92 (0.64, 1.19)	> 0.50	0.91 (0.64, 1.18)	> 0.50
Males, BMI (≥30 kg m^{-2} vs. 18.5–24.9 kg m^{-2})	1.06 (0.02, 2.10)	> 0.50	1.07 (0.04, 2.11)	> 0.50
Females, BMI (<18.5 kg m^{-2} vs. 18.5–24.9 kg m^{-2})	0.69 (-0.48, 1.86)	> 0.50	0.69 (-0.48, 1.86)	> 0.50
Females, BMI (25–29.9 kg m^{-2} vs. 18.5–24.9 kg m^{-2})	1.05 (0.63, 1.47)	> 0.50	1.01 (0.60, 1.43)	> 0.50
Females, BMI (≥30 kg m^{-2} vs. 18.5–24.9 kg m^{-2})	1.12 (0.31, 1.93)	> 0.50	1.08 (0.30, 1.87)	> 0.50
Males, diabetes mellitus (yes vs. no)	1.10 (0.56, 1.64)	> 0.50	1.09 (0.56, 1.62)	> 0.50
Females, diabetes mellitus (yes vs. no)	1.17 (0.55, 1.79)	> 0.50	1.18 (0.57, 1.79)	> 0.50

Notes: BMI, body mass index. DM, diabetes mellitus. CI, confidence interval estimated using the Wald statistics. POAG, primary open-angle glaucoma.

[1] *p* values of improvement in fit over the null model (with no trend in dose) are given, assessed via the Wald statistics.

[2] For all workers.

[3] For workers with neutron exposure.

Supplementary references:

1. Kuichi, Y. *et al*. Glaucoma in atomic bomb survivors. *Radiat. Res*. **180**, 422–430 (2013)
2. Kuichi, Y. *et al*. Association between radiation, glaucoma subtype, and retinal vessel diameter in atomic bomb survivors. *Sci. Rep*. **9**, 8642 (2019)
3. Little, M.P. *et al*. Occupational radiation exposure and glaucoma and macular degeneration in the US radiologic technologists. *Sci Rep.* 8, 10481, https://doi.org/ 10.1038/s41598-018-28620-6 (2018).
4. Preston, D., Lubin, J., Pierce, D. & McConney, M. Epicure Users Guide. (Hirosoft, Seattle, WA, 1993).
